# Study on anti-collapse performance of CFST structures under collision load in different cases

**DOI:** 10.1371/journal.pone.0317552

**Published:** 2025-02-12

**Authors:** Lian Song, Lin Wang, Xuan Wang

**Affiliations:** 1 College of Urban Construction and Engineering, Chongqing University of Arts and Sciences, Chongqing, China; 2 China MCC5 Group CORP. LTD., Chengdu, China; University of Zanjan, IRAN, ISLAMIC REPUBLIC OF

## Abstract

In view of the continuous collapse resistance of the remaining structure under impact column and side columns under vehicle loads, the composite plane frame of concrete filled steel tubes (CFST) was established by using the finite element software ABAQUS, and the orthogonal experimental design and analysis were carried out. Through numerical analysis, the whole process of continuous collapse of the structure caused by low-speed impact is simulated. The research results show that: in the collapse mechanism of the two working conditions, the deformation modes of the impact column and the remaining frame show two modes. According to the fact that the axial force of the adjacent bottom column of the non-impact column increases greatly at first and then tends to be stable after being impacted, the axial force of the non-adjacent bottom column fluctuates up and down around the impact front axle force. Comparing the two working conditions with the direct simulation method (DS) and the alternate path method (AP), the reliability of AP method in evaluating the internal force of the remaining structure under impact load is insufficient, and it is unsafe to judge the collapse degree of the structure. According to the result of range analysis, it can be seen that the velocity is the most important factor to cause the dynamic effect of the remaining structure in the two cases. This study deeply analyzes the influence of the structural damage caused by the low-speed impact of accidents on the continuous collapse of the remaining structures, which has very important theoretical and practical significance for improving the anti-continuous collapse ability of buildings.

## 1. Introduction

Concrete filled steel tubular (CFST) structure has many advantages under various loading conditions, so it is widely used in many high-rise and super high-rise buildings. However, during the service period, the CFST structure not only bears the normal design load, but also may bear the accidental impact load. For example, high-rise buildings are accidentally impacted by debris from crashed aircraft or hurricane flying rolls, bridges or offshore drilling platforms are impacted by ships, building structures or garages are accidentally impacted by cars, plant buildings are impacted by means of transportation, and the structure of the construction site is accidentally impacted by crane lifting heavy objects, etc. Scholars have also conducted relevant research, such as Sxa B and Yha B [1] introduced the performance of steel structure parking column under transverse impact, and studied the failure mode of impacted column; Yong Z and Hua Y [2] tested 9 CFST specimens with a drop hammer experimental device, carried out experimental and Numerical Research on their dynamic characteristics under transverse impact. From domestic and foreign research data, it can be seen that when steel reinforced concrete is used as a load-bearing structure for building columns, continuous collapse accidents caused by impact will result in huge losses of life and property.

On the one hand, scholars from all over the world have made some achievements in the study of progressive collapse of reinforced concrete frames and steel structure frames. McGuire W [3] and Burnett E [4] conducted the earliest research on the continuous collapse of building structures. Scholars such as Azim I [5], Dong F [6], Zhou [7] and Mucedero G [8] have mainly studied the factors affecting the collapse resistance of structures. Kl A and Zc A [9] take into account structural design parameters including structural load, geometry, material properties, and so on. In the existing codes [10], the design methods for structural resistance to continuous collapse can be mainly divided into four kinds: conceptual design method, tensile force method, member removal method and local reinforcement method. Amiri S [11], Mohamed [12], Gerasimidis S [13] and other scholars have studied the design methods of relevant codes against progressive collapse in terms of comparison and design. Zhong HH [14] studied the reliability of the progressive collapse resistance of steel frame structure under accidental load, and calculated the probability of progressive collapse of damaged structure and the reliability index of progressive collapse conditions. Huang YZ [15] found that the main design parameters have an important influence on the anti-progressive collapse characteristics of regular frame structures, and put forward a new fast algorithm for the collapse damage area, which provided an effective reference for the evaluation of the anti-progressive collapse performance of urban buildings. However, there are relatively few literatures devoted to the collapse mechanism of composite frames. At present, there is no specification on the collapse resistance design of composite frames.

At the same time, there are few experimental studies on continuous collapse of structures so far. Wang [16], Kong [17] and El-Desoqi M [18] have carried out the progressive collapse test, and studied the structural collapse analysis under the demolition column. Because of the high cost of non-repetition, and the difficulty of disaster load simulation and data collection, numerical simulation technology not only has strong problem-solving ability, but also can get effective data and new discoveries, which greatly expands the scope of collapse test and becomes an indispensable and effective way in the field of structural collapse. Dcfa B and Hrs B [19] put forward a numerical model for progressive collapse analysis of prestressed concrete beams and columns, and studied the progressive collapse behavior of prestressed concrete sub assemblies. Wang J X and Shen Y [20] studied the collapse resistance of all-welded beam-column structure with central column removed in fire scene by numerical simulation. Zheng L [21] takes the single-story 2 × 1 span CFST column-steel beam-reinforced truss floor slab system as the research object, and analyzes the continuous collapse resistance of the structure under the condition of removing the side columns. The force transfer law of beams and columns in the system is explored, and the vertical resistance provided by the bending mechanism of main girder, catenary mechanism of main girder and floor slab mechanism is quantitatively analyzed.

On the other hand, the current analysis methods of progressive collapse of building structures are generally not aimed at specific disaster types, and the research on progressive collapse resistance for specific disaster types mainly focuses on explosion and fire. Gqla B [22], Jahangiri V [23] and Shan S [24] have studied the progressive collapse analysis of structures under accidental conditions (such as explosions, terrorist attacks, fires). Because of the strong uncertainty of disaster load, the difficulty and complexity of this method are significantly improved. At present, there is no specific provision for this in design code, and there is little research on progressive collapse resistance under impact load.

Based on this background, it is of great theoretical and practical significance to study the progressive collapse resistance of CFST structure under lateral impact dynamic load by numerical simulation. In the early stage of this study [25], the alternate path method (AP) and direct simulation method (DS) have been used to compare and analyze the failure modes of the middle column under the impact load. AP method is an analysis method by assuming the sudden failure of a main stressed member of the structure, which is independent of the event causing the component failure and does not consider the type of accidental load. However, DS method needs to consider the impact mode of the impactor on the structure, and evaluate the progressive collapse resistance of the structure according to the dynamic response of the remaining structure.

According to the previous research results, it can be seen that even if the relatively accurate AP method is used for dynamic nonlinear analysis, it still cannot reliably and safely evaluate the resistance of structures to continuous collapse under impact loads, which is significantly different from the actual situation. Due to the neglect of the type of accidental load in the AP method, the failure process of columns is simplified, resulting in an overly simplistic consideration of the non-zero initial conditions generated by the structure and an overestimation of the continuous collapse resistance of steel-concrete frames under impact loads. In order to avoid sudden collapse of the entire structure, a comprehensive analysis of the load conditions should be conducted during the design process. For important building structures, it is necessary to evaluate the possible accidental load forms that may be suffered, so as to select appropriate analysis methods in a targeted manner, ensure the accuracy of the analysis results, and ensure targeted analysis. DS method starts with existing experiments and uses the general finite element program to model and calculate the lateral impact of steel columns and composite columns, verifying the effectiveness of the model. Secondly, under this premise, the numerical analysis of the entire process of continuous collapse of the structure was simulated by directly applying lateral impact objects to the bottom column. The research results indicate that the calculation results using the DS method are closer to the actual situation of structural collapse under impact load.

However, in the previous study, only one failure mode of the middle column was considered, while the other failure modes were not considered. Therefore, based on the application of the DS method, this study added the consideration of the collapse process of the side column failure mode to understand the collapse performance of the remaining structure under the side column failure mode. Through the analysis results, it is further obtained whether there is any difference in the dynamic response of the remaining structure under the impact condition of the middle column and the side column, so as to improve the structural collapse analysis of the frame structure when the impact load acts on different columns at the bottom. By discussing the dynamic response of composite frame structure under different impact conditions, the main influencing factors of structural progressive collapse are obtained, which provides suggestions for the design of structural progressive collapse resistance and ideas for further improving the design method of progressive collapse resistance.

## 2. Verification of finite element model

### 2.1. Introduction to impact test of preloaded components

**Fig 1:**
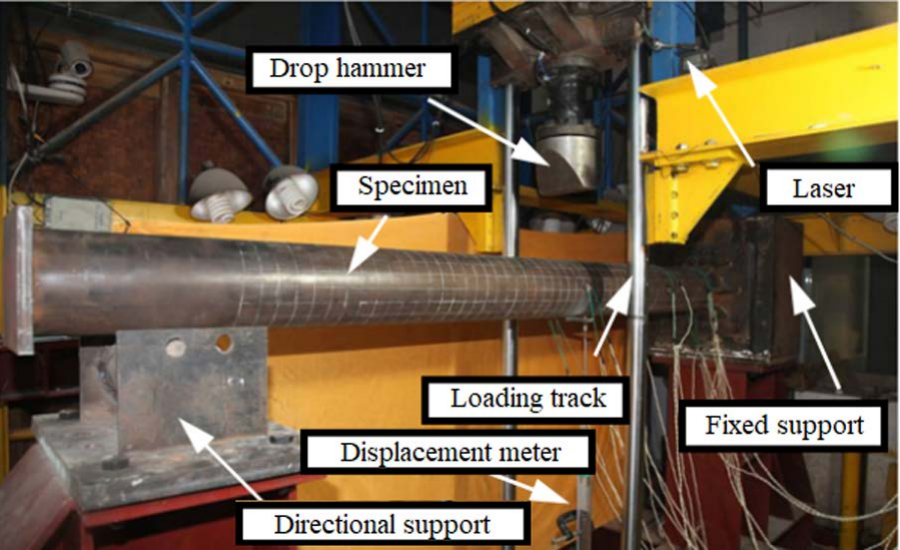
Impact test picture [ **26**].

Referring to the impact test of solid steel tube concrete components under preloading conducted by researcher [26], as shown in Fig 1, the key part of the test equipment is the drop hammer, which is accelerated by free falling from high altitude and converts potential energy into kinetic energy. During the test, the drop hammer produces a specified initial velocity and acts on the specimen. Through the impact process, the energy is transferred to the specimen, and after contacting for a period of time, it is separated from the specimen to produce rebound. In addition, the preloading axial force in the test is loaded by the butterfly spring group designed. At the moment when the specimen is deformed and shortened along the axial direction during the impact process, the spring releases the pre-stored elastic potential energy, so that the axial force is continuously loaded on the specimen to ensure that the axial force is not unloaded.

When making the test piece, firstly, empty steel pipes are processed, and the wall thicknesses of the steel pipes are 3.5 mm and 4.5 mm respectively, wherein the steel pipes with the thickness of 3.5 mm are straight welded coiled pipes, and the steel pipes with the thickness of 4.5 are seamless pipes, and the outer diameters of the adopted steel pipes are all 114 mm. Then pour concrete into it. When pouring concrete, first stand the steel pipe upright and place a steel plate on its lower part, and then pour concrete into the upper opening. Table 1 summarizes the detailed information of the experiment, where *D* represents the outer diameter of the steel pipe, *ts* represents the thickness of the steel tube, L represents the sample length, *V* represents the lateral impact velocity, and *M* represents the falling weight mass.

**Table 1:** Detailed information of testing specimens.

No.	Concrete	Boundary conditions	*D × ts × L (mm × mm × mm)*	*V (m/s)*	*M (kg)*	Pre-loading *(kN)*	Reference
**DHF42**	Filled	Fixed at both ends	○114 × 4.5 × 1200	7	229.8	215	[26]
**DHF44**	Filled	Fixed at both ends	○114 × 4.5 × 1200	7	229.8	450	
**DZF26**	Filled	Fixed at both ends	○114 × 3.5 × 1200	11.7	229.8	0	
**DZF31**	Filled	Fixed at both ends	○114 × 3.5 × 1200	11.7	229.8	200	
**DZF33**	Filled	Fixed at both ends	○114 × 3.5 × 1200	11.7	229.8	400	
**DZF34**	Filled	Fixed at both ends	○114 × 3.5 × 1200	4.4	229.8	200	

### 2.2. Axial force-impact coupling model

The methods of establishing finite element model include geometric model, boundary conditions and interface treatment, and the detailed information is described in reference [27]. This section focuses on the mesh size and axial force-impact coupling model. Regarding the mesh size, according to the references, when the ratio of the mesh size to the side length of the square beam section is less than 0.2, the calculation result is better. On the basis of referring to the suggested size ratio of 0.12, the calculated CFST members with continuously changing mesh size are tried. Finally, 1/40 of the circumference of the outer ring of the steel tube is selected for meshing, and the deformation is good. In order to simulate the axial force loading of the impact column more accurately, it is necessary to solve the axial force impact coupling problem and the numerical convergence of the model. The ABAQUS/Explicit model is adopted in the impact model, and the force of the amplitude curve is set smoothly to load the pre compression axial force smoothly until the axial force reaches a certain stable state. At the same time, as the drop hammer is in the gravity field in the model, the distance and initial velocity from the impact column can be obtained through the conversion of loading time, initial velocity and gravity acceleration, so that the impact of the initial impact velocity of the test impact will be ensured under the continuous loading of the preloaded axial force.

### 2.3. Result verification

The comparison between the following failure forms and the time history curves of the impact force (such as goodness of fit) shows that the numerical model can accurately reflect the whole process of the impact of the axial compression column under lateral impact load.

#### 2.3.1. Deformation mode.

**Fig 2:**
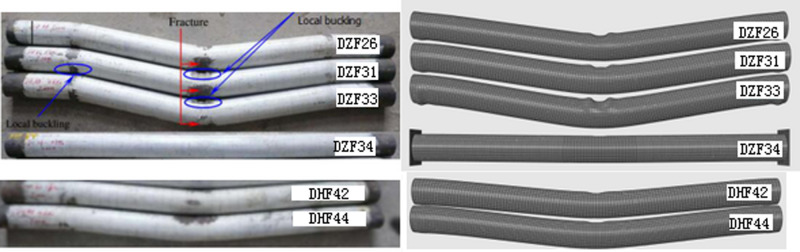
Failure modes comparison between tests and FEA.

During the impact process, the CFST specimen cracked at the bottom of the steel tube in the middle of the span, corresponding to the phenomenon of slow unloading in its impact time-history curve, but the impact energy has not been completely dissipated, so that under the action of residual impact energy, the steel tube cracks continue to extend upward along the axis of the steel tube, and at the same time, the concrete continues to crack, so that the bearing capacity in the middle of the span is continuously reduced until the impact energy is completely dissipated, at which time the impact force is also completely unloaded. Han LH [28] used hollow CFST members HCC and HSS for impact test. According to the comparative analysis of the test results, it can be concluded that the deformation mode of CFST column is different from that of hollow steel tubular column, in which the local deformation of CFST columns is different at the impact contact point, and the bulge caused by the filled concrete being partially crushed and expanded, which is obviously different from the impact section of hollow steel tubular columns being almost flattened. In addition, only when the impact energy is greater, the overall V-shaped bending deformation of CFST column will appear, as shown in Fig 2.

#### 2.3.2. Time history curve of impact force.

**Fig 3:**
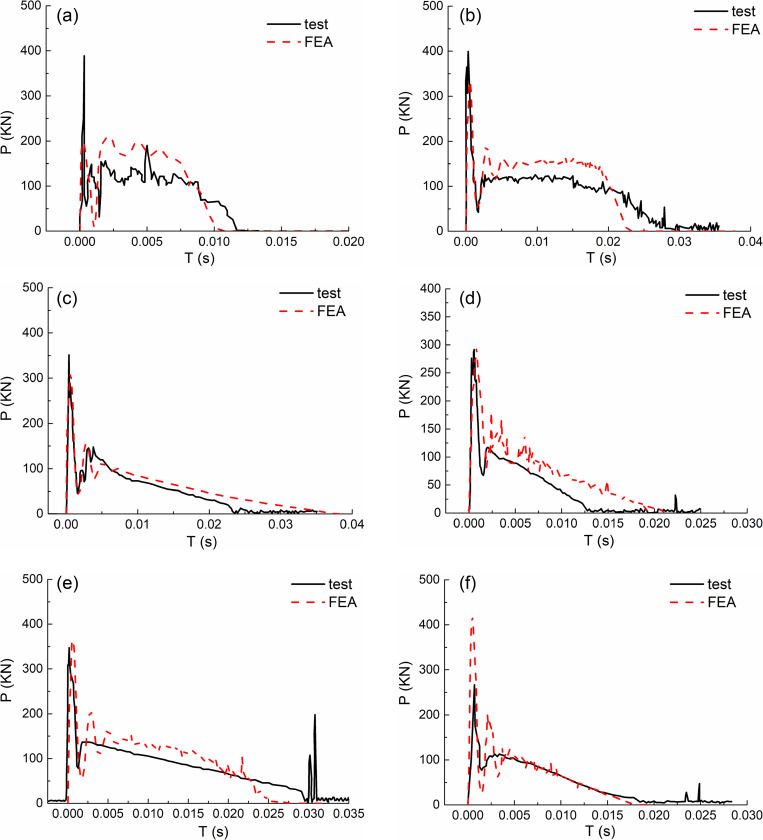
Impact force (P) versus time (T) curves. (a) DBF34. (b) DZF26. (c) DZF31. (d) DZF33. (e) DHF42. (f) DHF44.

As shown in Fig 3, by comparing with the test, the time history curves of the impact calculated by the finite element method are in good agreement with the measured curves, both in terms of trend and numerical value. It can be seen that all curves mainly go through three stages: oscillation stage, stability stage and attenuation stage.

## 3. FEA of progressive collapse for planar composite frame

### 3.1. Multiscale finite element model

#### 3.1.1. Analysis object.

**Fig 4:**
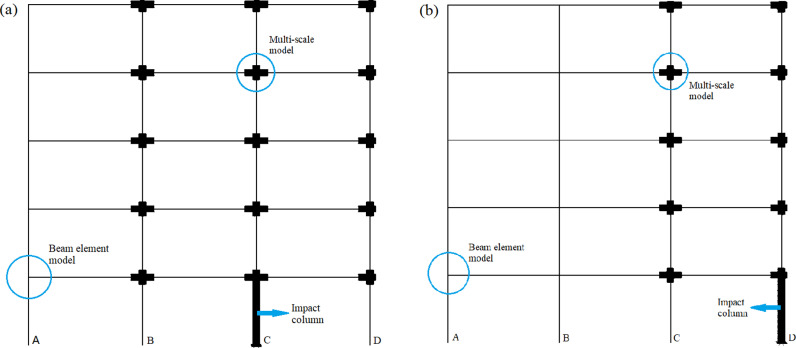
CFST plane frame structure model. (a) The impact middle column model. (b) The impact side column model.

According to the 5-story and 3-span CFST column-steel beam plane frame structure designed in the previous research, a multi-scale plane frame model with beam element, shell element and solid element is proposed, as shown in Fig 4. Beam-column connections are all welded and rigid. In view of the fact that the spatial structure form is not considered in the design of plane frame, a series of corresponding load values acting on the beam are obtained by setting the longitudinal span in a certain range for conversion. In order to make the structure tend to collapse, the vertical average wiring load with the same size on the beam is finally determined to be 45KN/m, and when checking the structure according to this load, it can meet the requirements of the limit state of bearing capacity and meet the design normal use conditions, and the thickness and shear strength of the beam-column joint area meet the requirements.

The basic information of the model: general information of the building: the column spacing is 6m and the floor height is 3.6m; Frame column: 360 ×  6mm (Section diameter ×  Steel pipe wall thickness); Frame beam: 300 ×  250 ×  8 ×  12 mm (High ×  Wide ×  Abdomen ×  Wing); Node design: the width of the reinforcing ring plate is 100mm and the thickness is 12 mm (the outer ring plate rigid joint); Material information: steel S355 is used for steel pipe and steel beam, and Es is 206GPa; Concrete C50.

#### 3.1.2. Column foot structure.

The common forms of column foot joints of CFST columns are: embedded, exposed and outsourced. In this paper, CFST column is embedded in the column foot joint of foundation beam connection, which is also a rigid joint. In modeling, in order to simplify the model, the column foot part is not established in detail, and it is equivalent to the column bottom of the ground elevation with complete consolidation constraint.

#### 3.1.3. Modeling method.

Considering that there is a floor on the frame beam of the actual engineering structure and the longitudinal surface of the frame column is also supported by the frame beam, this example restricts the out-of-plane movement of the frame beam. The gravity field is set in the model, and the external load does not include the self-weight of the structure. The actual external load is adopted, and the top surface of the frame beam is a linear load on the beam element, which is converted into a uniform surface load at the shell element in the refined node area. In DS method, the impactor is required to be restrained in all directions except the initial velocity direction to ensure the specified design velocity and impact position.

The method based on the multi-scale model of the structure can not only accurately simulate the real mechanical behavior of the structure, but also greatly reduce the workload and calculation. In this paper, a mixed multi-scale plane frame model with beam elements, shell elements and solid elements is established, as shown in Fig 4. In the macro model, the relatively macro beam element B31 is adopted for beams, columns and joints, and the element is divided into 300 mm.

The establishment of refinement model mainly includes the following three parts:

**(1) Refinement node:** Refined modeling area of joint: the steel pipe, steel beam and outer ring plate in the joint area adopt quadrilateral shell element (S4R). As they all belong to the same element type and have the same degree of freedom, in practice, as long as the welding quality is guaranteed, merge command is adopted between them to form an integral steel joint component, so as to reduce the setting of contact pairs, It can effectively improve the calculation efficiency of the model. The eight nodes hexahedral linear reduction integral element (C3D8R) is used for core concrete in the node area. The tie connection mode of binding constraint is adopted for the contact between joint steel pipe and concrete. Since the combined effect of the two has been considered in the constitutive relationship of core concrete, this method is in line with the reality.

**(2) Impact column:** For the column directly impacted in DS method, steel pipe and core concrete are regarded as two materials respectively, and the establishment of impact object. For the corresponding detailed modeling process, please refer to the method of simulating the lateral impact of column components in Section 2.3. The difference is that the speed direction of the vehicle here is not affected by the gravity field and maintains a constant initial speed before impact contact (the drop weight test is accelerated by free falling from high altitude); In addition, when the impact column end model is established, the column foot is treated as the same as the impact test column end connection according to section 3.1.2. However, the constraint of the upper end of the column is not simply supported, fixed or simply supported. Therefore, the actual situation of the impact column in the frame structure is considered by fine modeling of the impact column, and multi-point constraint method is used to deal with the connection interface between the macro model and the refinement model, so that the interface between the shell element, the solid element and the beam element can be connected and stressed together.

**(3) Connection interface:** The multi-point constraint method deals with the connection interface between the macro model and the refined model, so that the interface between the shell element, the solid element and the beam element is connected and stressed together, which is realized through the coupling function of the interaction command in ABAQUS. The connection at the interface is not to lose the degree of freedom of the macro model, but not to increase the extra constraints of the micro model as much as possible. Shi [27]introduced the connection technology of beam element model and shell element model, and proved the reliability of this connection. Because the shell element has out-of-plane rotational degrees of freedom, while the beam element node has three spatial rotational degrees of freedom, the transfer of rotational degrees of freedom between different scale models follows the principle of flat section. In ABAQUS, the coupling function of Interaction is used to connect the shell element with the beam element to bear the force together. The physical meaning of the coupling command ensures the coordination of the rotation between the connection section and the node and the lateral displacement of the beam.

**(4) Impact interface:** Surface-to-surface contact (Explicit) is adopted for the contact between the impactor and the impact column, the rigid body is set to contact the main surface, and the impact column is the contact slave surface. Panalty algorithm is used in the tangent direction, and the friction coefficient is 0; The normal direction adopts the Hard Contact algorithm, which means that the pressure perpendicular to the contact surface can be completely transferred when the master-slave surface contacts, and the contact pressure will be reduced to zero when the master-slave surface is separated. In addition, Tangential Behavior is used to consider the tangential bond stress between steel pipe and concrete surface, and the calculation formula suggested in reference [29] is shown in formula (1). Specifically, when the shear stress of the interface is less than $\tau_{bond}$ there is no relative sliding at the interface; When the shear stress of the interface exceeds $\tau_{bond}$ the bond will be destroyed and the interface will slide. At this time, the relative sliding of the interface is simulated by Columbia Friction Model, and the friction coefficient is 0.6.


$$\tau_{bond}=2.314-0.0195\cdot \left(D/t_{s}\right)$$ (1)

Where, $\tau_{bond}$——Bonding stress, in $N/mm^{2}$

*D*——the diameter of core concrete of circular cross-section member;

$t_{s}$——the wall thickness of the outer steel pipe.

#### 3.1.4. Material model.

In the frame model, there are three types of elements, beam element, shell element and solid element, and the other two materials, steel and concrete. When defining the material model of each solid component, the shell element and solid element adopt independent steel and concrete material model, and the column established by beam element adopts the unified material model of concrete filled steel tube, as follows:

**(1) Steel and concrete:** In the steel constitutive model that delicacy the node area, beam and failure column, the constitutive model and failure model of S355 steel which are used to simulate the fracture of impact column are cited in document [30], which is applied to the real stress-strain relationship curve of S355 material model in ABAQUS. When the strain rate effect of steel is taken into account, the effect of strain rate on the strength of steel is suitable for the dynamic problem of low strain rate. Therefore, the classical Cowper-Symonds model [29] is adopted. The shear damage model in the ductile metal damage command is used to simulate the fracture of steel pipe. This model includes the damage initiation criterion of the initial fracture point of material, and the parameters of the material when fracture occurs, including the fracture strain $\varepsilon_{s}^{pl}$ shear stress ratio, strain rate; The cumulative damage evolution rule is carried out according to the evolution rule of the material after fracture; The fracture displacement when the material is completely fractured is the product of the fracture strain and the unit size at this time. The specific values of the parameters are shown in Table 2.

**Table 2:** Material fracture parameters.

Initial fracture strain	Maximum shear stress ratio	Maximum strain rate (s^-1^)	Complete fracture strain
0.295	1.85	320	0.65

For the material model of the core concrete in the joint area and the failure column, the concrete constitutive relationship is the axial compression constitutive relationship model of the core concrete proposed according to [31], which is suitable for ABAQUS considering the mutual restraint relationship between them, and has been verified by the effectiveness of the simulation of this model by most scholars for the structure of CFST. The strain rate effect of concrete is based on the dynamic enhancement factor DIF and strain rate $(\dot{\varepsilon }=3\times 10^{-5}\sim 300s^{-1}$) of concrete according to CEB (1988) [32] of the European Union of concrete. The plastic damage model suitable for analyzing the damage properties of concrete under dynamic load is adopted in ABAQUS. The four important parameters in the model are usually taken as follows: ψ = 300 (expansion angle), c = 0.1 (potential function eccentricity), $K_{c}=2/3$ (ratio of the second stress invariant on the tension compression meridian), α = 1.16 (ratio of uniaxial compressive strength to biaxial compressive strength). In addition, the Poisson’s ratio of core concrete is 0.2, and the elastic modulus is based on the algorithm of concrete elastic modulus provided in ACI (ommittee318-05 (2005)) [33], $E_{c}=4700\sqrt{f_{c}}\left(\text{MPa}\right)$.

**Fig 5:**
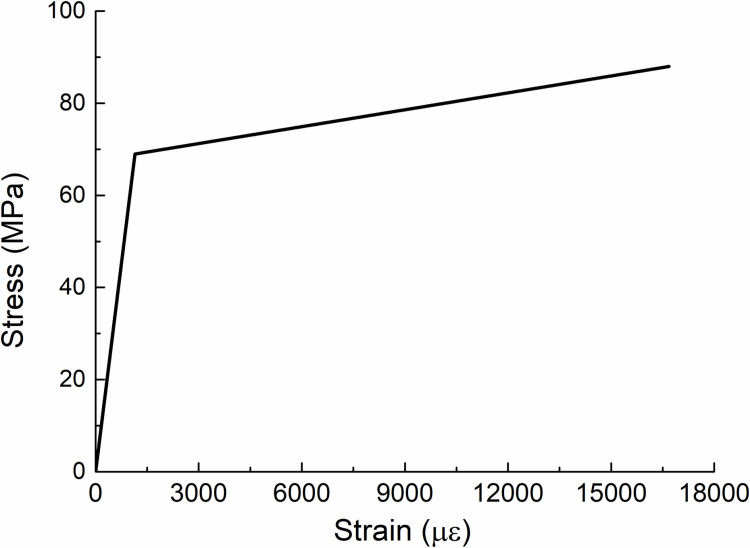
$\overset{}{\bar{\sigma }}-\varepsilon$ according to one material.

**(2) Unified material for concrete filled steel tube:** In the multi-scale modeling, the column established by beam element takes into account the fact that local effects need not be considered in practice. Therefore, steel pipe and concrete are regarded as a unity, which is composed of one material, and the performance characteristics of the unity can be evaluated by its overall geometric characteristics and each combination design index. The constitutive relationship of CFST materials based on unified theory is introduced below. The stress and strain curves of the CFST columns under axial compression are calculated by [34], for CFST short columns with circular section diameter of 360mm, steel tube wall thickness of 6mm, concrete C50 and steel S355, the calculated parameters are as follows: $f_{sc}^{y}=88\left(\text{MPa}\right)；f_{sc}^{p}=69\left(\text{MPa}\right),\varepsilon_{sc}^{p}=1144\left(\mu \varepsilon \right)$, thus $E_{sc}=f_{sc}^{p}/\varepsilon_{sc}^{p}=59999\left(\text{MPa}\right)；$$E_{sc}^{'}=1223\left(\text{MPa}\right)$. And the density of combination $\rho_{sc}=3136.5\left({\text{kg/m}}^{3}\right)$. According to the above calculation parameters, the stress-strain curve of CFST column under axial compression is simplified according to the Bilinear Isotropic Model, and the final σ -ε diagram is obtained (see Fig 5).

### 3.2. Orthogonal test design

The GSA2003 code in the United States specifies the location of the first floor for the removal of key components. Based on the study in this paper, the first story column failure is considered as the accidental load of vehicle impact. The calculation example in this paper is that the failure of the bottom side column D and the middle column C is considered because of the symmetry of the structure. For the convenience of subsequent description, the number of D (d) letter is used to indicate the working conditions of the bottom edge column impact, the same with the C (c) letter used to indicate the impact of the bottom column conditions. GSA Criterion for nonlinear analysis method, the ductility ratio of rotation and displacement of plastic hinge is taken as failure criterion, namely deformation criterion. The ultimate displacement value of the frame is calculated: the failure criterion with the limit angle value of 12 ° in GSA is adopted, that is, collapse occurs when the vertical displacement of the failure node is greater than 1275mm. Using orthogonal table $L_{25}\left(5^{6}\right)$ arrange the factor combination in this paper to simulate the corresponding impact conditions. According to 25 groups of tests in each case, the specific test scheme combination is shown in Table 3.

**Table 3:** Comparison of AP and DS methods of the middle column failure.

Orthogonal test (DS/AP > 1)	Axial force of column A	Axial force of column B	Axial force of column D	Node displacement
**Number**	18	19	19	14
**Ratio**	0.72	0.76	0.76	0.56

### 3.3. Comparative analysis

#### 3.3.1. Collapse mechanism.

**Fig 6:**
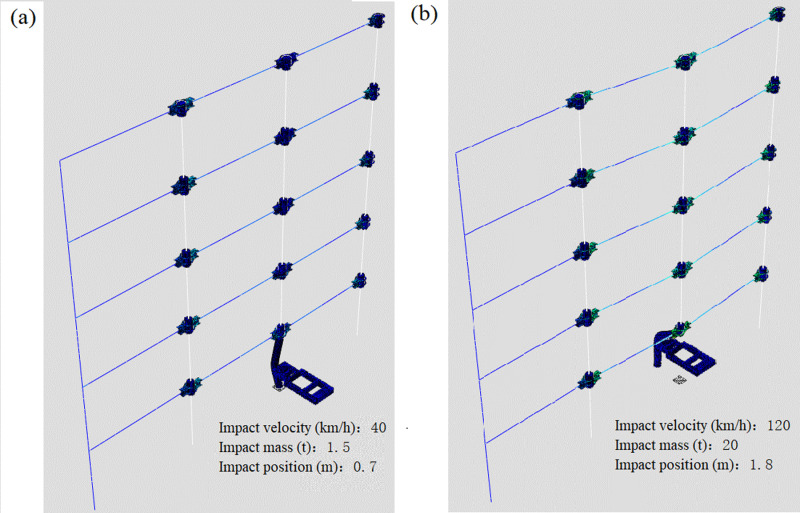
Frame deformation after impact on the middle column. (a) Model C1. (b) Model C25.

**Fig 7:**
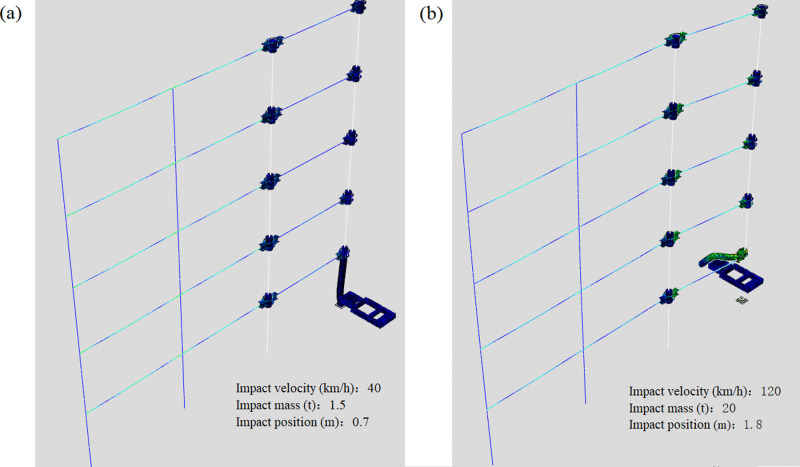
Frame deformation after impact on the side column. (a) Model D1. (b) Model D25.

According to the finite element calculation results of 25 groups of orthogonal tests of the middle column failure and the side column failure, it can be concluded that the deformation modes of the failed column can be divided into two types according to the impact degree under impact load. Among them, the most typical representative test groups C1, C25, D1 and D25 are selected to observe the overall deformation mode of the column. It can be seen from Figs 6 and 7 that the impact column has two deformation modes under impact load: local deformation and large deformation when the column base is damaged. Local deformation means that the bottom of the column is not damaged, and the overall bending deformation of the impact column occurs at the impact position. The entire impact process of the impact object has been completed within the analysis time, and a rebound effect has occurred; the large deformation when the column foot is damaged means that the impact column occurs. For the shear failure of the column foot, due to the large impact energy, the impact column will move together with the impacting object after the failure within the analysis time, resulting in a large displacement out of the overall plane. Observing the remaining structure also shows two modes: If the impact energy is low, only the small bending deformation of the impact column will be caused, the deformation of the other frame structure will have little influence; If the impact column fails, the vertical displacement of the node (failure node) at the beam will increase, and after the impact column is damaged, the internal force of the residual structure will redistribute, and the frame will finally appear a new equilibrium state.

#### 3.3.2. Axial force of non-impact column.

**Fig 8:**
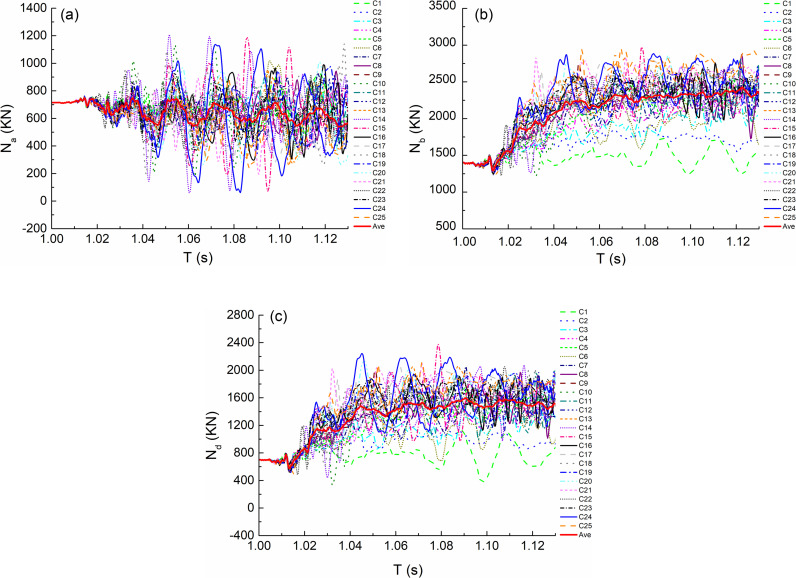
Internal force versus time (T) curves under condition C. (a) Axial force of A bottom column. (b) Axial force of B bottom column. (c) Axial force of D bottom column.

**Fig 9:**
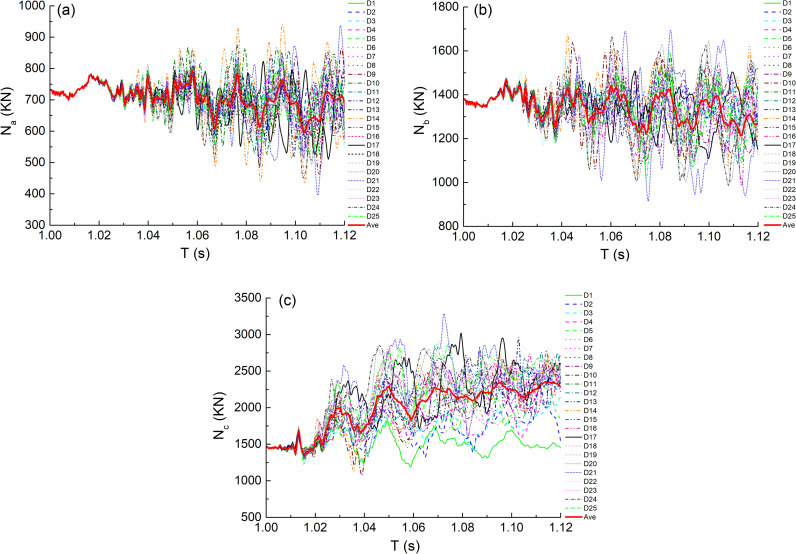
Internal force versus time (T) curves under condition D. (a) Axial force of A bottom column. (b) Axial force of B bottom column. (c) Axial force of C bottom column.

**Fig 10:**
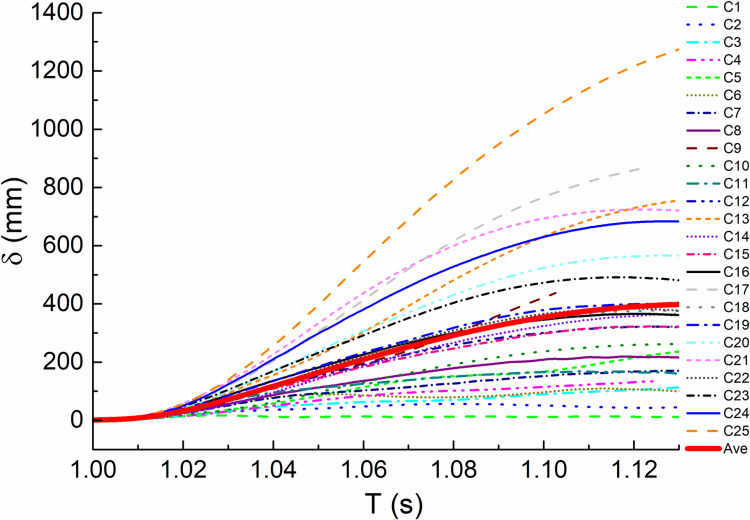
Vertical displacement of the failure column node versus time (T) curves under condition C.

**Fig 11:**
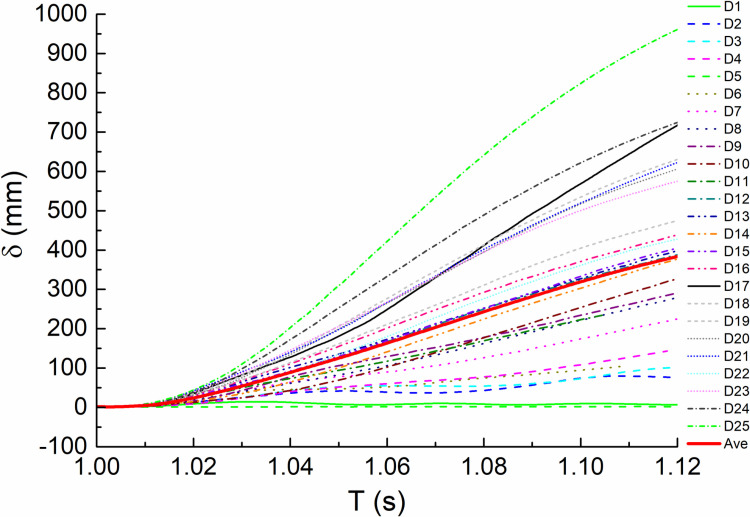
Vertical displacement of the failure column node versus time (T) curves under condition D.

Due to the failure of the impact column, the adjacent columns bear more axial force. Figs 8a–c and 9a–c respectively show the axial force time history curves of the adjacent bottom columns of the impact column in the analysis time under the impact conditions of the middle column and the side column, that is, under the condition of the middle column C, it corresponds to the adjacent columns A, B and D, and under the condition of the middle side column D, it corresponds to the adjacent columns A, B and C. In Figs 8–11, the numbers C(D)1-C(D)25 indicate 25 kinds of impact conditions when impacting the bottom center column (side column) according to the orthogonal test design, as shown in Table 2. Ave in the figure represents the average curve obtained by fitting the oscillation curve under 25 working conditions in each the figure.

**Fig 12:**
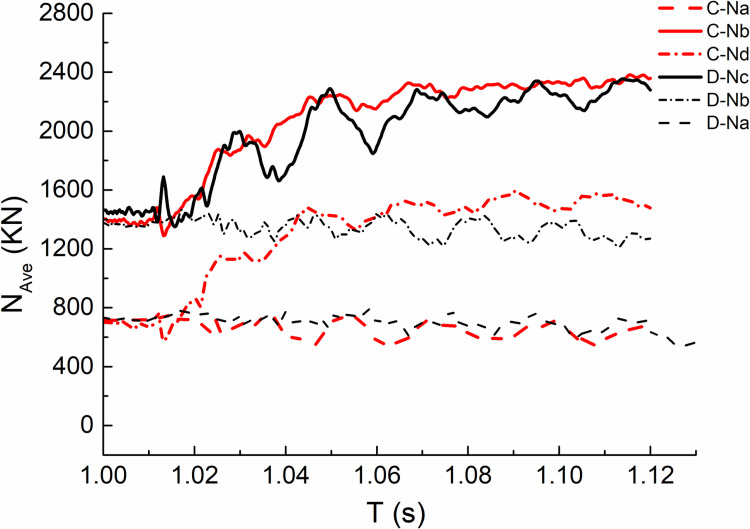
Average fitting internal force versus time (T) curves.

From the maximum value of oscillation curve, it can be seen that the axial force value of bottom column B under the impact condition of middle column is more unfavorable; Under the impact condition of side column, the axial force value of bottom column C is more unfavorable. The average distribution of orthogonal test results is observed from the average curve fitted by each oscillation curve, as shown in Fig 12, Where C(D)-N a(b/c/d) represents the average fitting axial force value of bottom column a(b/c/d) under the condition of C(D).The following laws can be obtained by observing the curve: the axial force time history curve of each bottom column is almost the same before column failure, and shows different development trends after the beginning of impact. For the adjacent bottom columns, the axial force first increases greatly and then tends to be stable; the axial force value of non adjacent bottom column fluctuates up and down around the axial force before impact.

#### 3.3.3. Displacement of failure point.

**Fig 13:**
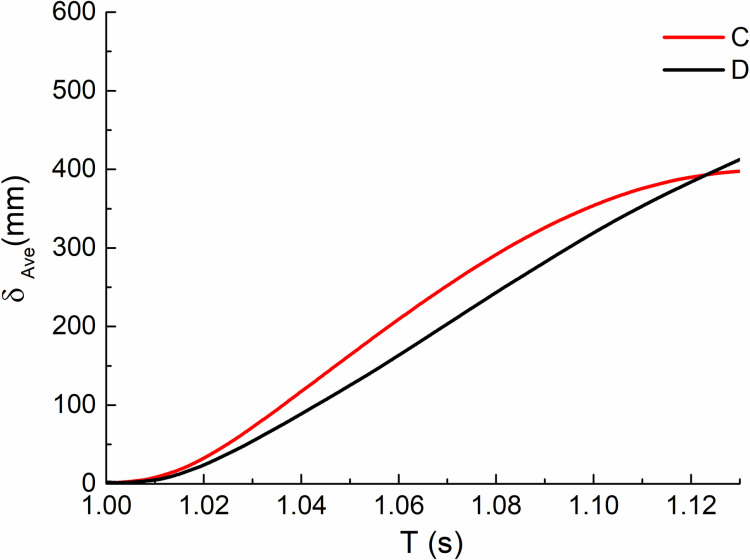
Average fitting vertical displacement of the failure column node versus time (T) curves.

Comparing the vertical displacement time history curves of impact column failure joints of all orthogonal test calculation models within the analysis time, as shown in Figs 10 and 11. It can be seen that the node displacement of each curve shows a trend of increasing first and then tending to be stable. When impacting the middle column, the most unfavorable combination is C25, and the displacement reaches 1274 mm, which is 1891 mm compared with D25 of the impact side column. The two are close to or even greater than the ultimate vertical displacement of the node calculated according to the collapse criteria, which indicates that the structure has collapsed. From Fig 13, we can observe the average fitting curve of each displacement under two working conditions, where C(D) stands for C(D) working condition.The maximum value (467mm, 408mm) is far less than the limit value, indicating that the structure can resist continuous collapse.

#### 3.3.4. Orthogonal test data analysis.

The data in Tables 3 and 4 show that the maximum values of each calculation index (bottom column axial force and node displacement) in DS method of orthogonal test are larger than those in AP method; the ratio indicates the ratio of the number of tests it exceeds to the total number of orthogonal tests 25. It can be seen that the ratio of internal force DS method to AP method of the remaining structure after column failure is greater than 1 is at least greater than 72%, which further proves that the AP method simplifies the dynamic effect of column failure on the remaining structure.

**Table 4:** Comparison of AP and DS methods of the edge column failure.

Orthogonal test (DS/AP > 1)	Axial force of column A	Axial force of column B	Axial force of column C	Node displacement
**Number**	24	25	19	9
**Ratio**	0.96	1	0.76	0.36

Because the main concern is the strong dynamic analysis of the impact process, and the analysis time is set to 130ms, the node displacement of some orthogonal tests has not reached the maximum stable value in this period of time. However, it can be seen from the table that the maximum displacement over this period of time has reached 36%, which is relatively conservative. However, when the middle column fails, the ratio of node displacement index exceeding AP method in orthogonal test reaches 56%. Especially for the side column failure condition, once the side column completely fails, its final vertical node displacement growth trend is divergent. Under the two working conditions, once the displacement of a node exceeds, not only the unfavorable situation will lead to the overall collapse of its local span, but also the ultimate excess ratio in quantity will increase greatly.

To sum up, if AP method is used to evaluate the reliability of the internal force of the remaining structure under impact load, it is obviously insufficient, and it is also unsafe to judge the collapse degree of the structure.

#### 3.3.5. Range analysis method.

In the orthogonal test data processing, the main concern is the maximum dynamic effect of the remaining structure. In order to get the impact degree of each factor on the frame, the range analysis method will be used for further analysis. According to the analysis results in sections 3.3.2 and 3.3.3, under two impact conditions, when N_max_ is the axial force value of the most unfavorable bottom column adjacent to C impact column and D impact column in each of 25 test schemes in the orthogonal table, the limit values in the analysis time are summarized in Table 5. In addition, in order to compare the influence degree of various factors (speed, mass, position) in the impact action, the axial force change parameter η of the above unfavorable column is introduced in Table 5 as the ratio of the axial force after impact to the axial force before impact. According to the principle of orthogonal design, when the velocity (mass, position) factor is at the ith level, the total influence degree of axial force of adjacent bottom columns of the test frame $K_{i}$:

**Table 5:** Summary of data analysis on transverse impact tests.

No.	Impact velocity (km/h)	Impact mass (t)	Impact position (m)	*N*_max_ (kN)	*η*
**C1(D1)**	40	1.5	0.7	1731.5 (1865.51)	1.23 (1.27)
**C2(D2)**	40	4.5	1.2	1917.88 (2321.03)	1.37 (1.59)
**C3(D3)**	40	7.5	1.5	2267.09 (2155.31)	1.62 (1.47)
**C4(D4)**	40	12	1.8	2424.66 (2446.39)	1.73 (1.67)
**C5(D5)**	40	20	2.5	2523.45 (2336.18)	1.80 (1.60)
**C6(D6)**	60	1.5	1.2	2443.77 (2070.21)	1.74 (1.41)
**C7(D7)**	60	4.5	1.5	2664.01 (2523.69)	1.90 (1.72)
**C8(D8)**	60	7.5	1.8	2872.14 (2658.79)	2.05 (1.82)
**C9(D9)**	60	12	2.5	2755.22(2662.01)	1.96(1.82)
**C10(D10)**	60	20	0.7	2687.57 (2649.25)	1.92 (1.81)
**C11(D11)**	80	1.5	1.5	2817.36 (2515.07)	2.01 (1.72)
**C12(D12)**	80	4.5	1.8	2654.23 (2758.85)	1.89 (1.89)
**C13(D13)**	80	7.5	2.5	2818.16 (2963.14)	2.01 (2.02)
**C14(D14)**	80	12	0.7	2703.8 (2738.52)	1.93 (1.87)
**C15(D15)**	80	20	1.2	2974.74 (2658.23)	2.12 (1.82)
**C16(D16)**	100	1.5	1.8	2760.26 (2792.7)	1.97 (1.91)
**C17(D17)**	100	4.5	2.5	2829.25 (3020.08)	2.02 (2.06)
**C18(D18)**	100	7.5	0.7	2724.21 (2906.5)	1.94 (1.99)
**C19(D19)**	100	12	1.2	2707.64 (2747.89)	1.93 (1.88)
**C20(D20)**	100	20	1.5	2859.36 (2835.74)	2.04 (1.94)
**C21(D21)**	120	1.5	2.5	2838.99 (3286.12)	2.02 (2.25)
**C22(D22)**	120	4.5	0.7	2692.18 (2578.87)	1.92 (1.76)
**C23(D23)**	120	7.5	1.2	2656.79 (2604.33)	1.89 (1.78)
**C24(D24)**	120	12	1.5	2884.8 (2851.73)	2.06 (1.95)
**C25(D25)**	120	20	1.8	2952.72 (2874.58)	2.11 (1.96)
*k*_**1*,c***_ ***(k***_**1*,d***_ **)**	1.55 (1.52)	1.794 (1.712)	1.788 (1.74)		
*k*_**2** *,d*_ ***(k***_**2*,d***_ **)**	1.914 (1.716)	1.82 (1.804)	1.81 (1.696)		
*k*_**3** *,c*_ ***(k***_**3*,d***_ **)**	1.992 (1.864)	1.902 (1.816)	1.926 (1.76)		
*k*_**4** *,c*_ ***(k***_**4*,d***_ **)**	1.98 (1.956)	1.922 (1.838)	1.95 (1.85)		
*k*_**5** *,c*_ ***(k***_**5*,d***_ **)**	2 (1.94)	1.998 (1.826)	1.962 (1.95)		
*R*_*c*_*(R*_*d*_)	0.45 (0.436)	0.204 (0.126)	0.174 (0.254)		

*k*_i_,_c_ *indicates the value of k*_i_ *under C working condition, and k*_i,d_ *indicates the value of k*_i_ *under D working condition; R*_c_ *is the r value under c condition, and R*_d_ *is the r value under d condition.*


$$K_{i}=\sum_{j=1}^{n}\eta_{ji}$$ (2)

Where, *η*_*ji*_ — the ratio value of velocity (or mass, position) factor for the jth test of frame at the ith level;

*n* — the number of occurrences of each level in 25 tests, *n* =  25/5 =  5, 1 ≤ *i* ≤ 5, 1 ≤ *j* ≤ 5.

The average value of velocity (or mass, position) factor at each level is obtained by *k*_*i*_,


$$k_{i}=K_{i}/n\left(i=1,2,3,4,5\right)$$ (3)

**Fig 14:**
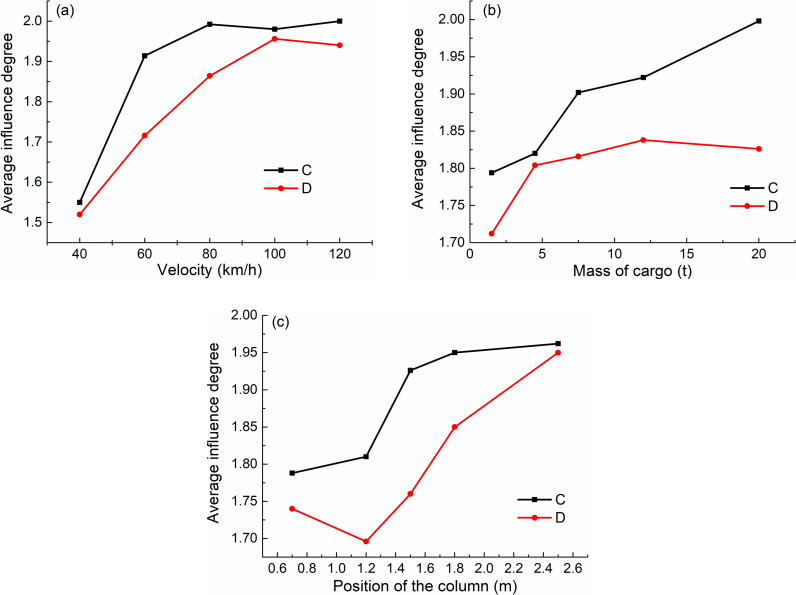
Single factor analysis. (a) Impact velocity. (b) Impact mass. (c) Impact position.

From Fig 14a, it can be concluded that for the failure of the center column, the velocity is in the range of 40~80 km/h, and when it increases significantly with the increase of velocity, the influence is small after it exceeds 80km/h; When the side column fails, the velocity is in the range of 40~100 km/h. With the increase of the velocity of the impactor, the influence degree of the average internal force increases linearly, and the influence of the velocity does not increase when it is more than 100 km/h.

It can be seen from Fig 14b that the impact of impactor mass on the average internal force increases linearly when the middle column fails, but the impact of mass is not as significant as that of the middle column when the side column fails, and the impact degree of load mass gradually increases with the increase of mass when it exceeds 5t.

From Fig 14c, it can be concluded that the impact point of the middle column is far away from the bottom of the column, and the influence degree of the average internal force is greater. However, for the side column, the impact point position only increases with the increase of the height position when it is higher than 1.2m, and it is more unfavorable when it is closer to the bottom of the column before it is lower than 1.2m. When the impact position is in the range of 0.8 ~ 2.6m, it has the greatest influence on the internal force of the remaining structure.

The parameter *R* value is the largest of the five numbers $k_{1}、k_{2}、k_{3}、k_{4}、k_{5}$ minus the smallest in the same factor, which reflects the variation range of test indexes when the velocity (or mass, position) factors fluctuate horizontally. The larger R is, the greater the influence of this factor on the test analysis index is, and vice versa. According to the magnitude of R, the primary and secondary order of factors can be judged.

For every factor (velocity, mass, or position), the parameter *R* value is the largest one of these five numbers minus the smallest one, which reflected the change with the level fluctuation of this factor. The *R* value is more larger, the influence factor is more important. According to the value of *R*, the order of the importance of the factors can be determined, and it can be seen that the most obvious factor is the velocity, as shown in Table 5.


$$R=\max\left(k_{i}\right)-\min\left(k_{j}\right)\begin{array}{cc} \left(i,j=1,2,3,4,5\right) & \end{array}$$ (4)

According to the comparison of the calculated results in Table 5, it is found that both the center column failure and the side column failure have the same speed, and the influencing factors are the most obvious.

## 4. Conclusion

The results of this study were compared with previous research, taking into account the dynamic effects of accidental loads on the structure. The full process analysis of the composite structure’s resistance to continuous collapse under impact loads was carried out, and compared and demonstrated with the existing dismantling method. The following conclusions were drawn:

(1) From the collapse mechanism, the deformation modes of the failed columns under the two working conditions show two modes: local deformation and large deformation when the column base is damaged; The remaining structure also shows two modes: the small deformation of the frame structure and the new equilibrium state of the frame caused by the destruction of the impact column.
(2) Under two impact conditions, the average fitting time-history curves of the positions of non-impact bottom columns and failure nodes are obtained by fitting the orthogonal test results. The axial force of adjacent bottom columns increases greatly at first and then tends to be stable, while the axial force of non-adjacent bottom columns fluctuates up and down around the impact front axle force.
(3) Compared with AP method, it can be obtained under both working conditions: if AP method is used to evaluate the internal force of the remaining structure under impact load, the reliability is obviously insufficient, and it is also unsafe to judge the collapse degree of the structure.
(4) According to the range analysis, it is proposed that the most obvious influencing factor under the two working conditions is the speed of each factor (speed, mass or position) of the impact effect.
(5) In this study, variable parameters can be used to further study, including material parameters (strength and stiffness of beams, impact columns and residual columns), structural layout parameters (column spacing, total number of stories, column height and lateral support effect), and load standard values.
